# Editorial: Targeted therapies for aggressive cancers

**DOI:** 10.3389/fcell.2023.1177266

**Published:** 2023-03-10

**Authors:** Sean B. Lee, Geoffrey Brown

**Affiliations:** ^1^ Tulane Cancer Centre, Tulane University School of Medicine, New Orleans, LA, United States; ^2^ School of Biomedical Sciences, Institute of Clinical Sciences, University of Birmingham, Birmingham, United Kingdom

**Keywords:** aggressive cancers, oncogenic targets, therapies, metasasis, relapse

During the past 40 years, the prevention and treatment of cancer have advanced substantially with ∼40% being preventable and ∼80% of patients surviving for 10 years or more. Despite these advances, two issues are longstanding and seemingly intractable. Whilst many cancers have a known aetiology (e.g., gene mutations and amplifications) there is not an effective treatment because effective inhibitors of cancer-specific targets are lacking. Second, the treatment of metastasized cancers has not advanced significantly. Often, patients are seen as beyond successful treatment. Cancer stem cells are largely the cause of metastasis and relapse and spared by conventional therapies. There is the need to find ways to eliminate these cells.

The Research Topic welcomed papers that addressed the treatment of intractable cancers and that described findings with the potential to be translated into new therapies. Initial considerations to the development of new treatments for intractable cancers are to identify a novel and appropriate target molecule for a particular cancer(s) together with the group(s) of patients that should benefit. For some time, there has been a move towards precision medicine based on the aberrant signatures of cancer cells. Three of the papers examine gene signatures to inform molecular guided therapies. There is then the need to find agents that will effectively target the molecule or the pathway of interest. The four paper examines the prospect to the clinical use of an inhibitor of tyrosine kinases to treat aggressive cancers.

The paper by Jiao et al. describes the outcomes from the open label, phase II Long March Pathway basket trial. This non-randomised trial consisted of several baskets. From 520 patients with intractable cancers, 115 had tier II gene alterations. Twenty-seven patients received targeted therapy that was based on molecular profiles. The disease control rate was 44.4% (12 patients), the overall response rate was 29.6% (8 patients), the median duration of response was 4.80 months, and the median progression-free survival was 4.67 months. From a molecular epidemiology study of 17, 841 Chinese patients with all types of cancer, the frequency of tier II gene alterations was 17.1%, with bladder cancer having the most alterations (26.1%), followed by breast cancer (22.4%), and non-small cell lung cancer (20.2%). For intractable cancers, the investigators concluded that molecular-guided therapies, *via* basket trials, provides significant clinical benefit.

The paper by Chen et al. focuses on neuroblastoma and examines ferroptosis-related gene signatures that associate with a poor prognosis. There are eight genes in the prognostic signature, namely, PROM2, AURKA, STEAP3, CD44, ULK2, MAP1LC3A, ATP6V1G2, and STAT3. There is differential gene expression between stages 1 and 4 neuroblastomas. All the signature genes were highly expressed in stage 1 neuroblastoma other than AURKA which might be a useful prognostic marker. The investigators have shown that the ferroptosis-related gene signature can be used to stratify patients into a high-risk and a low-risk group. In the case, the high-risk group had a lower overall survival than the low-risk group, revealing that perturbation to the processes relating to ferroptosis are more common within the high-risk group. Presently, it is unclear how ferroptosis-related signatures contribute to the development of neuroblastoma. Nonetheless, triggering ferroptosis may provide a future treatment for a subset of neuroblastoma patients.

Therapeutic efficacy of immunotherapy in breast cancer remains unclear and Cao et al. have sought to identify tumour antigens with a view to improving immunotherapy. The investigators screened for potential tumour antigens by examining the expression profiles for the invasive breast cancer cohort and their corresponding clinical data (from The Cancer Genome Atlas). The TIMER website was used to estimate the immune infiltration signatures. Expression of the tumour antigens CCNE1 and PLK1 was associated with poor prognosis and infiltration of antigen-presenting cells. The paper by Cao et al. has also examined how to identify patients who might benefit from immunotherapy. Immunogenic cell death shapes the environment that tumour cells reside in and activates the adaptive immune response. Thirty-four immunogenetic cell death-related genes are known to be associated with the improved survival of patients with lung, breast, and ovarian cancers. The investigators explored immunogenic death-related signatures revealing that they can be used to highlight response heterogeneity, predict prognosis, and identify patients that are suited to receiving combination treatment that includes immunotherapy based on the CCNE1 or PLK1 antigens.

The paper by Ma et al. examines CT053PTSA which is a tyrosine kinase inhibitor that targets various receptor tyrosine kinases (RTK), such as the cellular mesenchymal to epithelial transition factor (MET), AXL, which belongs to the belongs to the TAM family of RTK, vascular endothelial growth factor receptor 2 (VEGFR2), which regulates vascular endothelial growth factor-induced vascular endothelial function, FMS-like tyrosine kinase 3 (FLT3), and MERTK (another TAM-family RTK). These RTKs are all implicated in tumour pathogenesis. From *in vitro* cell lines studies, CTO53, the free-base form of CTO53PTSA, inhibited phosphorylation by the above kinases and blocked the actions of vascular endothelial growth factor and hepatocyte growth factor. Cell lines that had a high level of expression of MET were more sensitive to CT053. Tumour growth was significantly inhibited in cell line-derived (SNU-5 and MKN-45 gastric carcinoma cells) and patient-derived xenograft models (primary gastric cancer cells). Twenty patients were enrolled in a phase I trial of CT053PTSA and seventeen underwent tumour imaging with 29.4% showing stable disease. CT053PTSA was well tolerated, and based on this promising antitumour activity further trials are ongoing.

There is an increasing awareness of the need to develop treatments for cancers that are intractable. This is by no means an easy task. Basket trials guided by molecular profiling may well provide a good way forward. Driving the death of cancer cells by provoking ferroptosis is a topical area of interest and such can be guided by prognostic signatures. Whether the immune system of patients is responding sufficiently to immunotherapy to eliminate cancer cells and/or shape the tumour microenvironment is an avenue that is being explored to a large extent. Tyrosine kinase inhibitors, such as imatinib (Gleevec) and dasatinib (Sprycel) inhibit the tyrosine kinase activity of BCR-ABL1 which is the signature fusion gene for chronic myeloid leukaemia. Although imatinib and dasatinib have provided a targeted and mainline treatment for chronic myeloid leukaemia, the extent to which this success can be extended to intractable carcinomas is still being explored.

